# Supplementary material for: RELA∙8-Oxoguanine DNA Glycosylase1 Is an Epigenetic Regulatory Complex Coordinating the Hexosamine Biosynthetic Pathway in RSV Infection
Source: Cells. 2022 Jul 15;11(14):2210. doi: 10.3390/cells11142210 (PMC9319012; doi:10.3390/cells11142210)

Supplementary Figure S1: Overlap of N glycosylated peptides in two models of RELA knockdown

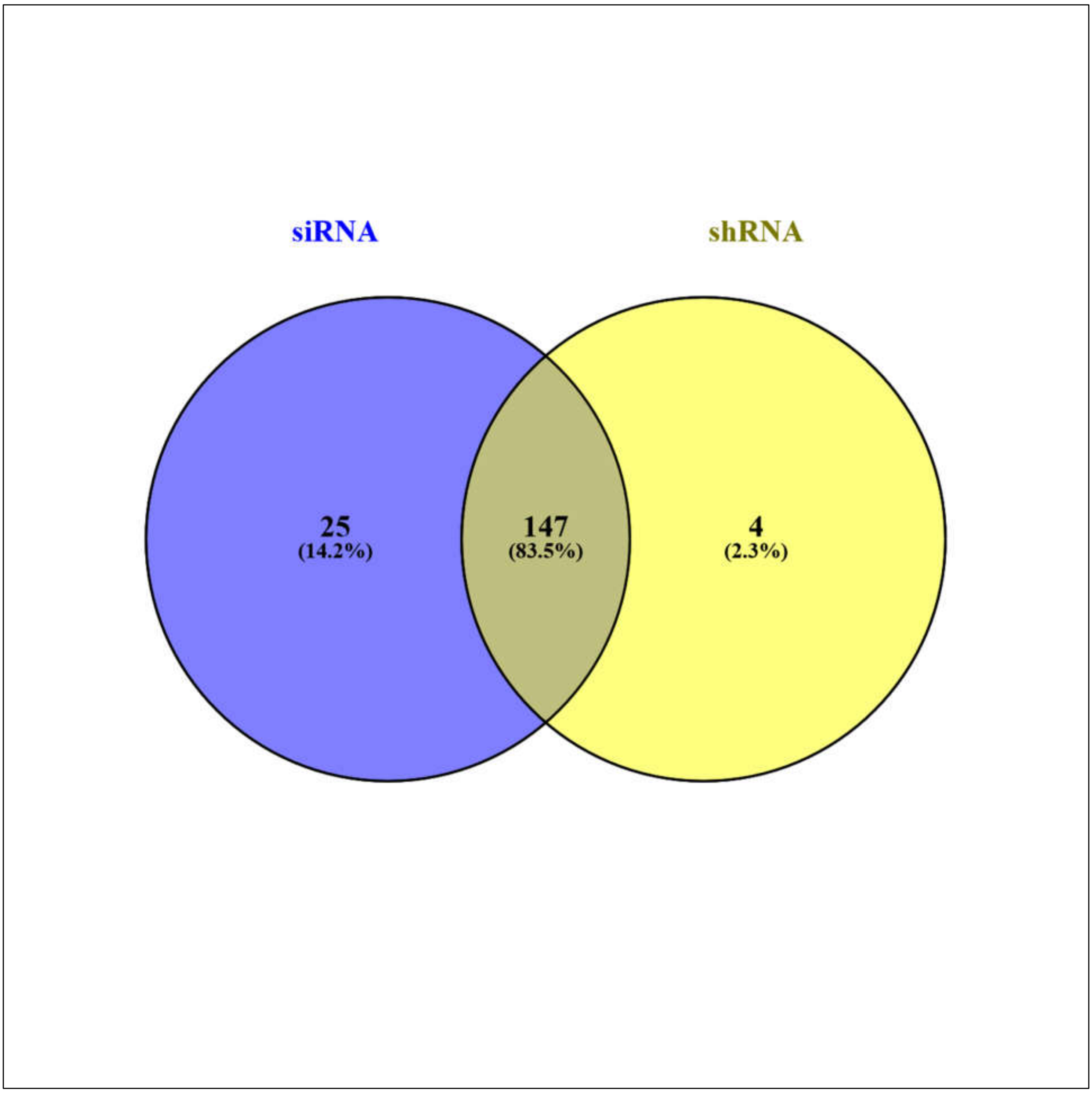

Supplementary Figure S2: Gating strategy for GFPT2 flow cytometry

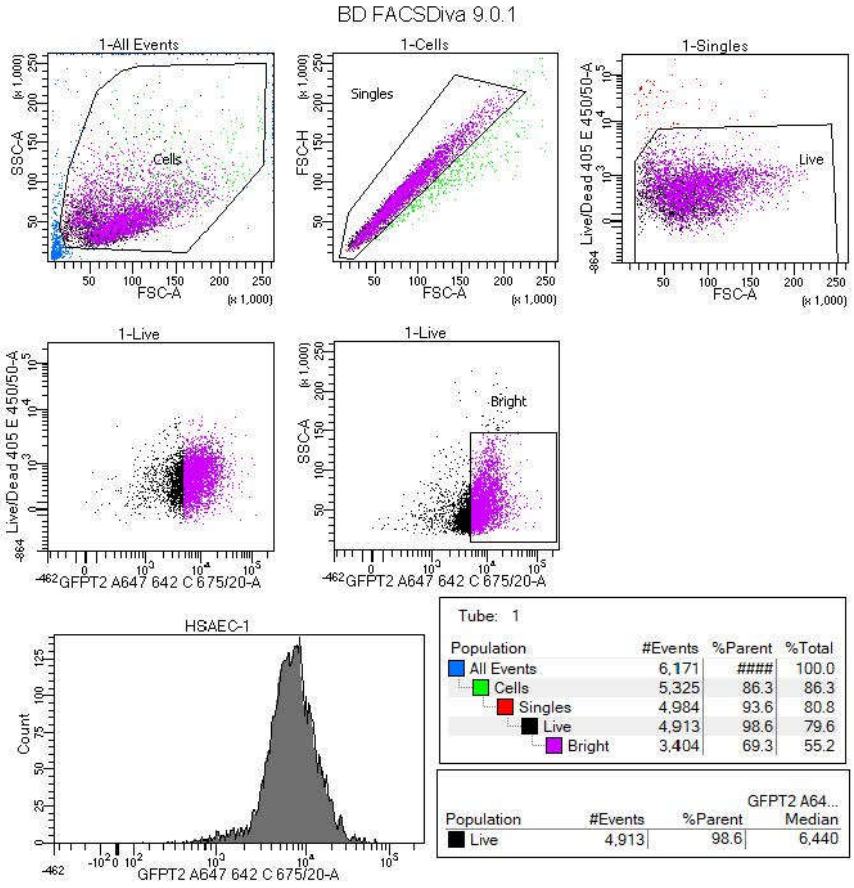

Supplement: Supplementary file 1 [file cells-11-02210-s001.zip › cells-1758367-supplementary.pdf]
